# Potential impact of parasites in the transmission of chronic wasting disease

**DOI:** 10.4103/NRR.NRR-D-24-01152

**Published:** 2025-04-29

**Authors:** Paulina Soto, Rodrigo Morales

**Affiliations:** Department of Neurology, The University of Texas Health Science Center at Houston, Houston, TX, USA; Centro Integrativo de Biología y Química Aplicada (CIBQA), Universidad Bernardo O’Higgins, Santiago, Chile

**Chronic wasting disease — a prion disease affecting cervids:** Many neurological conditions, including Alzheimer’s and Parkinson’s diseases, amyotrophic lateral sclerosis, frontotemporal dementias, among others, are caused by the accumulation of misfolded proteins in the brain. These diseases affect not only humans, but also animals. Prion diseases are a particular group of diseases among neurodegenerative disorders that can manifest in epidemic proportions in livestock and wild animals. These are fatal neurological conditions caused by the misfolding of a physiologically generated protein, termed cellular prion protein or PrP^C^, to a pathological isoform referred to as PrP^Sc^ (Prusiner, 1991). The events leading to the formation of PrP^Sc^ are not fully understood at the molecular level; however, these processes are known to occur either stochastically, be favored by mutations in the prion protein gene (*PRNP*), or templated by exogenous PrP^Sc^ particles. Prion diseases have been observed in various mammals, including humans. In all cases, these diseases are characterized by the progressive degeneration of the brain, leading to alterations in behavior, weight loss, and ultimately culminating in death.

Chronic wasting disease (CWD) is a prion disease affecting cervids. Although most animal prion diseases affect farmed animals, CWD affects both captive and free-ranging populations. Its increasing dissemination in North America has made it a pressing concern. The origin of CWD remains a topic of ongoing research. While some theories suggest either spontaneous origins or transmission from animals infected with scrapie they have not yet been conclusively proven. In North America, CWD prevalence is significant, reaching up to 30% in free-ranging populations and as high as 80%–90% in some captive populations. Initially confined to northeastern Colorado and southeastern Wyoming, CWD has now expanded into a large part of North America, highlighting its growing impact across the continent. Since its first report on a captive mule deer in Colorado nearly 50 years ago, CWD in North America has spread to various regions. By March 2025, this disease has been identified in 36 states of United States and 4 Canadian provinces (National Wildlife Health Center, 2025). Outside of North America, CWD was first reported in South Korea in 2000. This particular event was linked to imported infected farmed elk from Canada. More recently, CWD-infected animals have been identified in free-ranging reindeer and moose in Norway, Finland, and Sweden. The rapid spreading of this disease, and the multiple contributing factors participating in its propagation, make CWD one of the most contagious prion diseases.

As of now, there is no cure for CWD, nor are there any pharmacological treatments that can alleviate the disease. The primary method for controlling its spread is by identifying cases through diagnostic tests and removing affected or at-risk animals. Currently, the only approved diagnostic tests for CWD are immunohistochemistry and enzyme-linked immunosorbent assays. However, these tests are primarily limited to animal-derived and *postmortem* samples. The development and optimization of prion detection methods that can accurately identify CWD in tissue and environmental samples, while also allowing for non-invasive, early CWD diagnosis, is crucial. The development of new generation prion detection methods is expected to significantly impact the control of CWD.

**Figure 1:**
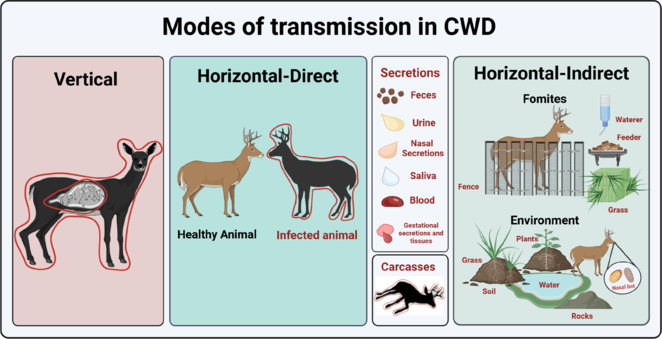
Mechanisms involving the transmission of chronic wasting disease (CWD). Schematic representation of vertical and horizontal mechanisms of CWD transmission. For horizontal transmission, direct (animal-to-animal) and indirect (through contaminated fomites) contacts are represented. The roles of potentially relevant animal components (tissues and excreta) in direct and indirect contacts are also represented. Created with BioRender.com.

**Mechanisms of chronic wasting disease transmission:** CWD prions are thought to be transmitted horizontally and vertically (**Figure 1**). Empirical evidence indicates that the offspring from CWD-positive dams are at higher risks to display the disease. Furthermore, prions have been documented in the fetal, gestational, and reproductive tissues of infected deer (Bravo-Risi et al., 2021). Regardless, horizontal transmission is acknowledged as the most effective way of CWD spreading. The latter is proposed to occur through direct animal-to-animal contact, or indirectly by contaminated environmental fomites. Specifically, direct contact involves the exposure of naïve animals with infected subjects through bodily fluids, excreta, or tissues. Indirect contact refers to the animals’ interactions with components of the environment such as water, soil, plants, or other biological or inert objects (Bartz et al., 2024). Considering the above-mentioned elements as potential vectors of CWD transmission, the spread of this disease is favored by the susceptibility of cervids to get infected by the oral route.

Multiple evidence indicates that CWD prions persist in the environment for several years, particularly in soils. This is majorly due to the strong adherence of prions to some soils (Bartz et al., 2024). This underscores the long-term environmental impact of CWD, as organisms living in contact with soils (e.g., plants, earthworms, and insects) may be exposed to the prions bound on them. In turn, the same organisms may contribute to the spread of these infectious particles. Moreover, other animals sharing environments with CWD-infected animals are expected to be exposed to infectious prions, helping in their dissemination (Pritzkow, 2022). All these factors are thought to have a pivotal role in CWD’s endemic prevalence and extensive geographic distribution. Nevertheless, the mechanisms of prion spread between animals and across geographic regions are not yet clear, as well as what is the hierarchical role of the different elements of the environment in prion dissemination and transmission.

**Figure 2:**
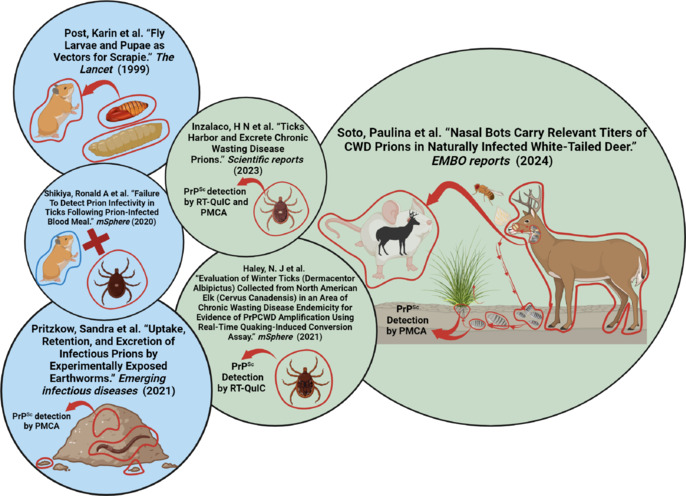
Current reports describing the potential role of invertebrates in the transmission of chronic wasting disease (CWD). The figure illustrates relevant studies assessing the potential role of invertebrates as vectors or spreaders of infectious prions. Studies involving experimental prion exposure to parasites are depicted in a blue background, while those using specimens collected from naturally CWD-infected hosts are marked with a green background. Created with BioRender.com.

**Invertebrates as potential vectors of chronic wasting disease spread:** As mentioned, the contact between healthy cervids and contaminated environmental components is acknowledged as one of the main causes of CWD transmission (Otero et al., 2021). Recent evidence provided by our group and others suggests that invertebrates may play a relevant role in these processes. Unfortunately, this is an emerging area of research, and so, just a handful of studies have explored the potential relationship between parasites and CWD transmission. Some of this evidence is discussed below and is summarized in **Figure 2**.

An early report, focused on a different animal prion disease (scrapie), studied mites collected from farms housing prion-infected sheep. The authors concluded that these parasites harbor infectivity as demonstrated in mouse bioassays (Wisniewski et al., 1996). Although this data indicated that mites could potentially act as transmission vectors for scrapie, the high attack rate for a sheep-to-mouse transmission, and the use of an antibody that does not recognize the mouse prion protein, flawed the conclusions posited by the authors. An additional experimental study evaluated the potential role of larvae and pupae of carnivorous flies as prion vectors (Post et al, 1999). These invertebrates were shown to carry prion infectivity after being fed with the brain of scrapie-infected hamsters, suggesting that prion diseases can be mechanically transmitted by these insects.

Considering the extensively described recalcitrance of prions in soils, experiments were conducted with earthworms to evaluate their potential to disseminate prions from infectivity foci (Pritzkow, 2022). There, it was found that earthworms can attach to, absorb, accumulate, and disperse infectious prions in artificially contaminated soils. This study also showed that the waste excreted by these worms contained relatively large quantities of PrP^Sc^. These findings indicate that earthworms exposed to prions mechanically bind and spread infectious particles. However, considering the experimental nature of these findings, their relevance in natural settings is still contentious.

To fill the gaps mentioned above, recent research describes relevant findings applied to naturally infected cervids. Specifically, these latest reports have focused on the interactions between CWD-infected cervids and parasites. These studies are potentially relevant considering that these animals are afflicted by a wide variety of parasites in free-ranging and captive environments (Lupi, 2005). In addition, it has been shown that prion infectivity is present in multiple tissues and fluids relevant for parasitic colonization (e.g., blood, nasal secretions, muscles, feces, etc.). White-tailed deer serve as hosts for various external and internal parasites. Arthropods, such as ticks and nasal flies, affect the Cervidae family by acting as parasites that use their hosts to grow and develop. The distribution of parasites affecting cervid families depends on the geographical location, environmental factors, and seasonal time. White-tailed deer (*Odocoileus virginianus*) can be affected by numerous ectoparasites, including at least 19 species of ticks, *Cephenemyia phobifer* flies, and other arthropod types. Ticks are blood-sucking parasites that feed on cervids for up to 14 days and grow on blood meals up to 100 times their original weights. Research on ticks and their potential role in spreading CWD has produced conflicting results. One study, using *D. Andersoni* nymphs, found that the experimental exposure of these parasites to prion-infected Syrian hamsters or blood from the same animal species was ineffective in transmitting the disease and lacked *in vitro* prion seeding activity (Shikiya et al., 2020). However, more recent studies involving winter ticks (*Dermacentor albipictus*) and black legged ticks (*Ixodes scapularis*) indicate that these parasites, when exposed to CWD prions in both experimental and natural scenarios, can carry *in vitro* prion seeding activities as evaluated by PMCA and RT-QuIC (Haley et al., 2021; Inzalaco et al., 2023). At present, there are no reports describing the specific infectivity titers carried by CWD-exposed ticks, so their role in disease transmission is still unclear.

**Nasal bots as potential vectors of chronic wasting disease transmission:** Nasal bots are relevant parasites affecting multiple cervid species. Part of their developmental cycle occurs in the nasal cavity of the host, an anatomical structure of relevance for CWD. The *Oestrinae* subfamily of nasal flies naturally affects mammals. They use the nasal cavities or the mouth as an entrance gate and then move into the host. *Cephenemyia phobifer* flies parasitize white-tailed deer, mule deer, and elk. The larvae of these flies, in their first stage, are most frequently found in groups located in the retropharyngeal pouches of the host. The larvae develop through their second and third stages in this place, using the animal’s secretions or blood as food. When the larvae reach the third stage, they are expelled by the host through the mouth or nose into the environment. While in the soil, the cuticle of these parasites hardens. Depending on environmental conditions and temperature, they emerge as flies after 15 to 24 days. Following mating, the female flies seek a suitable host to deposit their eggs, thus completing the reproductive cycle (Soto et al., 2024).

The study “Nasal bots carry relevant titers of CWD prions in naturally infected white-tailed deer” (Soto et al., 2024) provides evidence that parasites from non-clinical, CWD-infected white-tailed deer contribute to the transmission and environmental contamination of this disease. Our study revealed that nasal larvae exposed to infected deer carry PrP^Sc^. Furthermore, we demonstrated that these parasites have substantial quantities of prions to transmit CWD. The latter is relevant as this study showed, for the first time, prion infectivity in a naturally exposed parasite. Importantly, our estimations suggest that the prions present in a single bot collected from a non-clinical, CWD-infected deer are enough to infect another deer. Furthermore, our research demonstrated that nasal larvae can amass prions irrespective of the time and the prion exposure dosage. Additionally, the study demonstrated the role of these parasites as environmental pollutants as they can spread their prion cargo to soil and plants. Overall, the data included in this study suggested that these nasal larvae play multiple roles in the transmission and spreading of CWD.

**Conclusions and perspectives:** Multiple reports from the last few years have provided relevant information regarding the ecology of CWD. Specifically, we now better understand how prions enter the environment, and how they are dispersed and become available to naïve subjects (**Figure 1**). In this perspective, we discussed published evidence on how parasites interact with CWD prions and their potential roles as disease vectors. Importantly, our recently published article described, for the first time, the presence of prion infectivity in parasites collected from naturally infected animals (Soto et al., 2024). As communicated in our article, we estimate that one nasal bot is more than enough to infect a deer if ingested. Then, how could deer get in contact with these fly larvae? Deer are ruminant animals that feed mostly on plant materials. The consumption of plants is linked with the ingestion of significant amount of soils, especially when plants are scarce and more difficult to collect. Additionally, deer have been described as ingesting soil to incorporate minerals from it and combat the harmful effects of some plants. Since fly larvae are expelled by their hosts and further develop in soils, it is reasonable to assume that naive deer could come into contact with these parasites. How? Prions are resistant to degradation and avidly bind to soils. So, a plausible scenario is that dried, pulverized bots or bot shells may become part of the materials ingested by deer. Considering that most of the infectivity seems to be located in the bot shell (Soto et al., 2024), the complete development of these parasites into flies may not have major consequences for the contamination of soils. It is important to note that a single cervid can host more than a hundred of these parasites at a single time. The fact that the parasites tested in our experiments were collected at some point of the long and silent prion incubation period makes this scenario worth of further analyses.

As part of their life cycle, nasal bots eventually turn into flies. Considering this, it is important to determine whether adult flies carry CWD prions and if eggs released by them are able to transmit disease to naïve animals by oronasal routes.

Throughout history, various parasites have been described as essential vectors to spread of multiple infectious diseases. However, the interactions between prions and parasites as CWD vectors remain poorly understood. It is essential to fill this knowledge gap because if parasites play a role in CWD transmission, there could be opportunities to control it. That is why our recent findings demonstrate the necessity for conducting a more comprehensive study and characterization of parasites affecting animals vulnerable to CWD.

*We apologize for the many missing references that should also be quoted. Several review articles have been listed for further reading*.

*This work was supported by a grant from NIH (R01AI132695) to RM*.
